# FLP Recombinase-Mediated Site-Specific Recombination in Silkworm, *Bombyx mori*

**DOI:** 10.1371/journal.pone.0040150

**Published:** 2012-06-29

**Authors:** Ding-Pei Long, Ai-Chun Zhao, Xue-Jiao Chen, Yang Zhang, Wei-Jian Lu, Qing Guo, Alfred M. Handler, Zhong-Huai Xiang

**Affiliations:** 1 State Key Laboratory of Silkworm Genome Biology, Institute of Sericulture and Systems Biology, Southwest University, Chongqing, China; 2 USDA/ARS, Center for Medical, Agricultural and Veterinary Entomology, Gainesville, Florida, United States of America; Auburn University, United States of America

## Abstract

A comprehensive understanding of gene function and the production of site-specific genetically modified mutants are two major goals of genetic engineering in the post-genomic era. Although site-specific recombination systems have been powerful tools for genome manipulation of many organisms, they have not yet been established for use in the manipulation of the silkworm *Bombyx mori* genome. In this study, we achieved site-specific excision of a target gene at predefined chromosomal sites in the silkworm using a FLP/*FRT* site-specific recombination system. We first constructed two stable transgenic target silkworm strains that both contain a single copy of the transgene construct comprising a target gene expression cassette flanked by *FRT* sites. Using pre-blastoderm microinjection of a FLP recombinase helper expression vector, 32 G3 site-specific recombinant transgenic individuals were isolated from five of 143 broods. The average frequency of FLP recombinase-mediated site-specific excision in the two target strains genome was approximately 3.5%. This study shows that it is feasible to achieve site-specific recombination in silkworms using the FLP/*FRT* system. We conclude that the FLP/*FRT* system is a useful tool for genome manipulation in the silkworm. Furthermore, this is the first reported use of the FLP/*FRT* system for the genetic manipulation of a lepidopteran genome and thus provides a useful reference for the establishment of genome manipulation technologies in other lepidopteran species.

## Introduction

Site-specific recombinase (SSR) technology is an important molecular biotechnology that was developed during the 1980s. Through the genetic manipulation of the eukaryotic genome and exogenous DNA by SSR-mediated recombination between two recombination target sites (RTs), SSR can induce the replacement, inversion and tissue-specific knockout of target genes [1]. SSR can overcome the disadvantages of other types of recombination technology, such as homologous recombination and transposon-mediated recombination; such disadvantages include low efficiency and random integration without targeting. As a result, this technology has gradually been widely applied to many areas of transgenic organism research, particularly for the genetic engineering of higher eukaryotic organisms [2]–[6].

Currently, the most commonly used SSR systems are Cre/*loxP* from *Escherichia coli* phage P1 [7], FLP/*FRT* from the 2-µm plasmid of *Saccharomyces cerevisiae* [8] and ФC31/*att* from the *Streptomyces* phage ФC31 [9]. Since the first report of the application of the Cre/*loxP* system for generating a tissue-specific knockout mice model [10], this system has been widely used to study gene function in mammalian cells and to construct transgenic mouse models of disease [2], [11]. Because the recombination catalyzed by ФC31 integrase (Int-ФC31) between the heterotypic sites *attP* [34 base pairs (bp) long] and *attB* (39-bp long) is directional and irreversible [12], Gao *et al.* developed an efficient site-specific integrase-mediated repeated targeting (SIRT) method to target a single locus repeatedly and to facilitate targeted mutagenesis in *Drosophila* [3]. The ФC31/*att* system has also had an important role in the integration of transgenes into the mammalian genome and for the development of gene therapy [13], [14], [15]. As a member of the integrase or tyrosine-based family of SSR technologies [16], the FLP/*FRT* system has also emerged as a powerful tool to manipulate genomes of transgenic plants, mammals, insects and other higher eukaryotic model organisms. In recent years, the FLP/*FRT* system has been widely used in *Arabidopsis thaliana* [17], rice (*Oryza sativa*) [4], mouse (*Mus musculus*) [5], *Drosophila melanogaster* [18], *Caenorhabditis elegans* [6] and other higher eukaryotic organisms, to achieve gene knockouts, gene knock-ins, point mutations, deletion mutations, genomic large fragment deletions and other genetic engineering operations.

FLP recombinase can identify specifically *FRT* sites (FLP recombination target site) and mediate site-specific recombination reactions between two identical *FRT* sites [19], [20]. The position and relative orientation (i.e. same or opposite direction) of the two *FRT* sites determine the outcome (i.e. insertion, excision, inversion or reciprocal translocation) of the FLP recombinase-mediated recombination reaction [21]. In plants, the FLP/*FRT* system has been studied and used extensively for genome modification. FLP recombinase has been shown to catalyze site-specific excision of selectable marker genes from various transgenic plant species, including tobacco (*Nicotiana tabacum*) [22], maize (*Zea mays*) [23], rice [4] and other plants. In recent years, the FLP/*FRT* system has also been used for gene knockout and conditional gene activation in mammalian cells and transgenic mice [5], [24]. It has also been widely used to generate genetic mosaics in soma and germlines [25], chromosome rearrangements [26] and large fragment deletion mutations in the *Drosophila* genome [18]. Both the FLP/*FRT* and Cre/*loxP* systems have been jointly applied to investigate the function of two genes at the same location in the *Drosophila* genome [27]. In *Drosophila*, a strategy called FLP recombinase-mediated cassette exchange (FLP-RMCE) has been developed to avoid genomic position effects, which often confounds direct comparison of allelic transgenes [28]. FLP-RMCE methodology lends itself to a variety of approaches both in basic and applied research, including the fast generation of producer clones based on a previously characterized genomic site and an increase in the efficiency of site-specific integration [28], [29]. Although the recombination activity of FLP recombinase has also been demonstrated in other insect systems, such as mosquito (*Aedes aegypti*) embryos [30] and in cultured cells and embryos of the silkworm (*Bombyx mori*) [31], its application is still very much lacking in insects compared with other organisms.

The silkworm, *Bombyx mori*, is a useful model lepidopteran that has been domesticated and reared for silk production for more than 5000 years [32]. It is currently one of the most important economic insects worldwide, particularly in developing countries such as India, Brazil and China, where sericulture is still a major economic resource for farmers, with important social impacts. After the completion of a draft map [33], [34], fine map [35] and resequencing of different mutant *B. mori* genomes [36], the functional genomics of this organism has become one of the main areas of research into this species. To facilitate functional genomic research of the silkworm as a model system for Lepidoptera, it is necessary to establish different silkworm functional genomics research tools, including site-specific genetically modified mutants. However, there has been no progress in developing the technology for site-specific insertion and knockout in the silkworm genome. SSR technology is an effective and target-specific methodology used for functional analysis of genes and genetic transformation, but there are only a few reports of its use in the silkworm. As far as we are aware, the only published reports are on the use of the FLP/*FRT* system in silkworm by Tomita *et al.*, who used the system to achieve site-specific excision of an extrachromosomal (plasmid-based) gene-containing DNA fragment in silkworm cells and embryos [31], and by Nakayama *et al.*, who used the ФC31/*att* system to achieve site-specific recombination between *attB* and *attP* sites in cultured silkworm *Bm*N4 cell lines [37]. So far, SSR technology has yet to be established for the genome manipulation of silkworms.

In this report, we first describe a method using the FLP/*FRT* site-specific recombination system to achieve site-specific excision of a target gene at a predefined target site in transgenic silkworm genome. We demonstrate that injection of an FLP recombinase-expressing vector can induce site-specific excision in transgenic silkworms. This methodology will facilitate the development and application of the FLP/*FRT* system for the genetic manipulation of the silkworm and the potential use of this system for the analysis of silkworm gene function.

**Figure 1:**
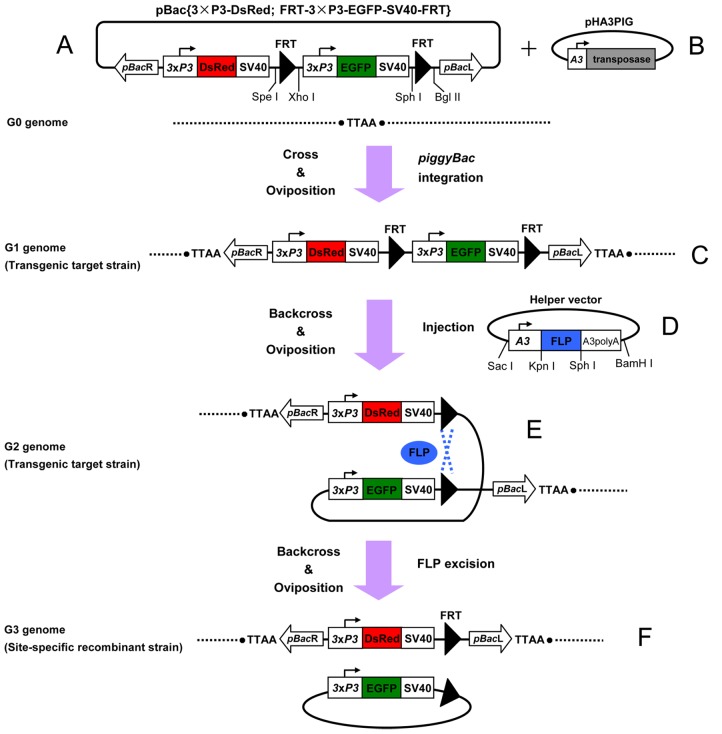
Strategy for FLP recombinase-mediated site-specific recombination in silkworms. The *piggyBac*-derived vector pBac{3×P3-DsRed; FRT-3×P3-EGFP-SV40-FRT} (A) was inserted into the TTAA site of the G0 silkworm germ cell genome to produce a stable G1 transgenic target strain (TTS) (C) mediated by *piggyBac* transposase derived from plasmid pHA3PIG (B). The TTS was transgenic for a 3×P3 promoter-driving DsRed gene (*red box*) expression cassette and a cassette that was flanked by two 48-bp *FRT* sites (*black triangles*) in the same orientation. A 3×P3 promoter-driving EGFP gene (*green box*) was placed internally to the two *FRT* sites. Site-specific recombination between the two *FRT* sites of G2 TTS germ cell genome (E), mediated by FLP recombinase derived from helper vector pSLA3-FLP (D), result in the deletion of the 3×P3-EGFP expression cassette from the genome of G3-positive site-specific recombination strain (SSRS) individuals (F). 3×P3, 3×P3 promoter; SV40, SV40 polyadenylation signal sequence; A3, silkworm cytoplasmic actin 3 promoter; A3 polyA, polyadenylation signal sequence of silkworm A3 gene; *pBac*L, left arm of *piggyBac* transposon; *pBac*R, right arm of *piggyBac* transposon. The restriction enzyme sites for the construction of recombinant vectors are shown.

## Materials and Methods

### Experimental Animals

The Chinese lineage *B. mori* bivoltine inbred strain Dazao has been maintained in our laboratory. It was necessary to change the diapause character of Dazao eggs for DNA pre-blastoderm microinjection. The 15°C-IMES germline transformation strategy used for the Dazao strain was based on the report of Zhao *et al.* [38]. After being injected, the eggs were maintained at 25°C in a moist chamber (85–90% relative humidity) until hatching. The larvae were reared at 25°C (75–80% relative humidity) and fed with mulberry leaves.

### Construction of Vectors

#### Recombinase-mediated site-specific recombination target construct

The *piggyBac*-derived vector pBac{3×P3-DsRed; FRT-3×P3-EGFP-SV40-FRT} (3×P3, 3×P3 promoter; DsRed, red fluorescent protein; EGFP, enhanced green fluorescent protein; SV40, SV40 polyadenylation signal sequence) (Figure 1A) was constructed as described below. A 1.3-kb 3×P3-EGFP-SV40 fragment was amplified by PCR from pBac{3×P3-EGFPaf} [39] with the primer pair 3×P3-F-XhoI (5′-tatactcgagGTTCCCACAATGGTTAATTCG-3′) and SV40-R-SphI (5′-gactgcatgcTACGCGTATCGATAAGCTTTAAG-3′). The amplified fragment was double-digested with *Xho*I and *Sph*I, and inserted between the *Xho*I/*Sph*I site of the plasmid pSLfa1180fa [39] to generate pSL{3×P3-EGFP-SV40}. Two 48-bp *FRT* fragments were obtained by annealing the following sequences of the oligonucleotides: FRT-SpeI/XhoI-F (5′-ctagtGAAGTTCCTATTCCGAAGTTCCTATTCTCTAGAAAGTATAGGAACTTCc-3′) and FRT-SpeI/XhoI-R (5′-tcgagGAAGTTCCTATACTTTCTAGAGAATAGGAACTTCGGAATAGGAACTTCa-3′); FRT-SphI/BglII-F (5′-cGAAGTTCCTATTCCGAAGTTCCTATTCTCTAGAAAGTATAGGAACTTCa-3′) and FRT-SphI/BglII-R (5′-gatctGAAGTTCCTATACTTTCTAGAGAATAGGAACTTCGGAATAGGAACTTCgcatg-3′). The annealing conditions were as follows: initial denaturation at 94°C for 5 min; reduced by 1°C per 90 sec until 25°C; 25°C for 5 min; and then stored at 4°C.

The two 48-bp *FRT* fragments were inserted between the *Spe*I/*Xho*I and *Sph*I/*Bgl*II sites of the plasmid pSL{3×P3-EGFP-SV40}. The plasmid pSL{FRT-3×P3-EGFP-SV40-FRT} was generated. pBac{3×P3-DsRed; FRT-3×P3-EGFP-SV40-FRT} was then constructed by cloning a 1.3-kb *Asc*I fragment from pSL{FRT-3×P3-EGFP-SV40-FRT} into *Asc*I cut pBac{3×P3-DsRedaf} [40].

#### FLP recombinase expression construct

The FLP recombinase-expressing helper vector pSLA3-FLP (Figure 1D) was constructed as described below. A 0.65-kb silkworm cytoplasmic actin 3 gene promoter (A3 promoter) fragment was amplified by PCR from pHA3PIG (Figure 1B) [41] with the primer pair A3-F-SacI (5′-tatcgagctcATGCGCGTTACCATATATGGTG-3′) and A3-R-KpnI (5′-tataggtaccCTTGAATTAGTCTGCAAGAAAAG-3′). The amplified fragment was digested with *Sac*I and *Kpn*I, and inserted into the plasmid pSLfa1180fa to generate pSL-A3. The 0.37-kb silkworm A3 polyadenylation signal sequence (A3 polyA) was PCR-amplified from Dazao genome with an A3 polyA-F-SphI (5′-gcatgcatgcAGGAAGTGCTTCTAAGCGT-3′) and A3 polyA-R-BamHI (5′-gcatggatccGTGCTCCTAGCGTAACTGTC-3′) primer pair. The PCR product was digested with *Sph*I and *Bam*HI, and cloned into the plasmid pSL-A3 to generate pSL-A3-A3 polyA. The FLP recombinase gene was amplified by PCR from the plasmid pKhsp82-FLP [28] with the following primer pair: FLP-F-KpnI (5′-gatcggtaccATGCCACAATTTGGTATAT-3′) and FLP-R-SphI (5′-gatcgcatgcTTATATGCGTCTATTTATGTAGG-3′). The 1.28-kb PCR product was inserted into the *Kpn*I/*Sph*I site of pSL-A3-A3 polyA to generate pSLA3-FLP.

The above sequences that are underlined show the restriction enzyme cutting sites. The sequences of the PCR products and resulting recombinant plasmids were confirmed by sequencing.

### Production of the Target Transgenic Silkworm Strain

Plasmid DNA for pre-blastoderm microinjection was purified using a QIAGEN Plasmid Midi Kit (Qiagen, Hong Kong, China), and the prepared DNA solution was stored at –20°C until being used. pHA3PIG was used as the helper plasmid for the production of *piggyBac* transposase. According to the 15°C-IMES germline transformation strategy [38], we collected the G0 non-diapause eggs from strain Dazao within 2 h following oviposition for microinjection. A 1∶1 (volume ratio) mixture of the 450 ng/µL pBac{3×P3-DsRed; FRT-3×P3-EGFP-SV40-FRT} vector and 400 ng/µL helper plasmid pHA3PIG in super-pure water were injected into each egg with a FemtoJet 5247 microinjector system (Eppendorf, Hamburg, Germany), and each egg was injected with 5–10 nL of the mixture. The injection hole was sealed with non-toxic glue (Instant Strong GlueMini, Japan) and the G0 embryos were allowed to develop at 25°C. G0 adults were mated with each other or backcrossed with the wild-type Dazao strain.

The expression of the red fluorescent protein (DsRed) and enhanced green fluorescent protein (EGFP) in G1 embryos, larvae, pupae and adults was detected using an Olympus MacroViewMVX10-AUTO fluorescent stereomicroscope (Olympus, Tokyo, Japan) with a RFP or GFP filter, respectively. Filters passing light between 510 and 550 nm for DsRed, and between 460 and 490 nm for EGFP were used for excitation. The individuals with DsRed- and GFP-positive G1 offspring were identified as germline-positive transgenic silkworms. G1-positive larvae from different broods were reared (with each brood being a unit), and the FLP/*FRT* system transgenic target strains (TTSs) were then produced.

### Determination of the Insertion Position and Copy Number of TTS Transgene Constructs

Genomic DNAs were extracted from G1 TTS moths and wild-type moths (as controls). DNA was purified using an improved phenol/chloroform method after proteinase K treatment [42]. Genomic DNA (approximately 10 ug) was digested with *Hae*III and circularized by overnight ligation at 16°C using T4 DNA ligase (Promega, USA). The ligated DNA was treated with phenol/chloroform and then precipitated with ethanol. Approximately 50–100 ng ligated DNA was used as a template for inverse PCR. Primers were used to recover the flanking sequence of the *piggyBac* transposon as described by Ding *et al.* [43]. For the 5′ junction (*piggyBac* left arm), the forward primer PLF (5′-CTTGACCTTGCCACAGAGGACTATTAGAGG-3′) and reverse primer PLR (5'-CAGTGACACTTACCGCATTGACAAGCACGC-3′) were used. For the 3′ junction (*piggyBac* right arm), the forward primer PRF (5′-CCTCGATATACAGACCGATAAAACACATGC-3′) and reverse primer PRR (5′-AGTCAGTCAGAAACAACTTTGGCACATATC-3′) were used. PCR was performed as follows: initial denaturation at 95°C for 5 min, then 35 cycles of 95°C for 30 sec, 63°C for 45 sec and 72°C for 3 min, followed by 72°C for 10 min.

PCR fragments were separated by electrophoresis in a 0.8% (w/v) agarose gel. Each single band was picked up from the gel and purified using a gel extraction kit (Omega, USA). The purified fragments were cloned into the plasmid pMD19-T simple and sequenced with the M13F/R primer to identify the boundary sequence of the insertion site.

Sequencing results were analyzed using NCBI BLAST searches (www.ncbi.nlm.nih.gov) and the silkworm genome database [SilkDB (http://www.silkdb.org/silkdb/)]. Localization of the silkworm genomic insertion sites of the *piggyBac*-derived vector was completed using the SilkMap application (www.silkdb.org/silksoft/silkmap.html).

**Table 1:** Injection of *piggyBac*-derived vectors in G0 silkworm embryos of the strain Dazao.

Injected vector	Number ofInjectedeggs	Number ofhatchedeggs (%)	Numberof fertilemoths	Numberof G1broods	Numberof broodswith DsRedand GFP-positivelarvae	Numberof DsRedand GFP-positive G1larvae inthe broods	Percent ofG1 broodswith DsRedand GFP-positive larvae (%)
pBac{3×P3-DsRed;FRT-3×P3-EGFP-SV40-FRT}+pHA3PIG	330	119 (36.06%)	40	28	2	28	7.14

**Figure 2:**
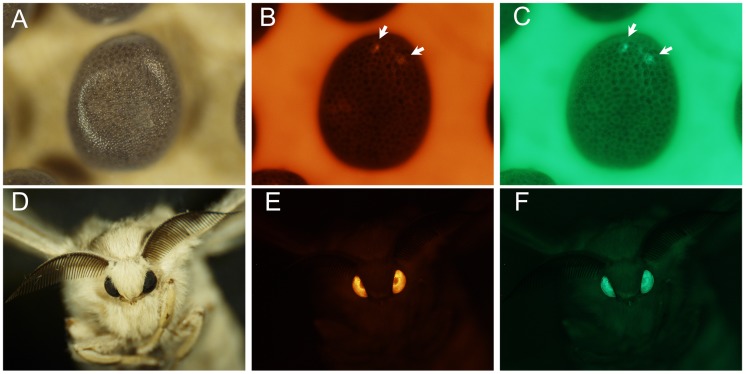
Expression of the DsRed and EGFP genes in TTS silkworms. (A–C) show white light (A), RFP-fluorescent (B) and GFP-fluorescent (C) images of 6-day-old G1 TTS-1 embryos. Arrowheads denote the position of the RFP and GFP fluorescence. (D–F) show white light (D), RFP-fluorescent (E) and GFP-fluorescent (F) images of the G1 TTS-1 adults.

### Injection of the pSLA3-FLP Vector into Embryos and Detection of Positive Site-specific Recombinant Silkworms

pSLA3-FLP was used as the helper plasmid for the production of FLP recombinase. Heterozygous G1 FLP/*FRT* system TTS adult males were backcrossed with the female adults of the wild-type Dazao strain (treated with 15°C-IMES [38]) to produce a G2 line of non-diapause embryos, heterozygous for the transgene, for microinjection. Microinjection of the pSLA3-FLP helper plasmid (325 ng/µL) was completed following the procedure described above. G2 larvae were reared at 25°C and fed with mulberry leaves. The G2 adults with DsRed- and GFP-positive phenotypes were selected and backcrossed to adults from the wild-type Dazao strain. Six-day or seven-day-old G3 embryos were screened for DsRed and EGFP expression in the larval nervous system and ocelli using the fluorescent stereomicroscope equipped with appropriate filters. Only DsRed-positive recombinant G3 individuals lacking EGFP expression were reared to adulthood and sib-mated to generate offspring. Finally, the FLP recombinase-mediated site-specific recombination strains (SSRSs) of transgenic silkworm were established.

### Analysis of Positive Site-specific Recombinant Silkworms

#### PCR analysis

The primer pairs P–F (5′-TACGGCGCGCCAAGCTTAAGGTGCA-3′) and P–R (5′-AATTCGAATGGCCATGGGACGTCGA-3′) were used to confirm individuals from FLP recombinase-mediated SSRSs of transgenic silkworms. The extracted genomic DNA from G1 TTS adults, G3 SSRS adults and wild-type adults were used as the template for PCR. The purified PCR fragments were cloned into the plasmid pMD19-T simple and sequenced with the M13F/R primer to identify the sequence of the FLP recombinase-mediated excision site.

#### Southern blotting analysis

25 µg genomic DNAs (G1 TTS adults, G3 SSRS adults and wild-type adults) were fully digested with *Xho*I and *Bgl*II, and separated by electrophoresis in 0.8% (w/v) agarose gel. DNAs were transferred directly onto nylon filters (Hybond N+, Amersham Bioscience) and immobilized by incubation for 30 min at 120°C. The probes were prepared as follows: a 720-bp EGFP fragment was amplified by PCR from pBac{3×P3-EGFPaf} with the primer pair pEGFP-f (5′-ATGGTGAGCAAGGGCGAGG-3′) and pEGFP-r (5′-CTACTTGTACAGCTCGTCCATGCCG-3′). A 678-bp DsRed fragment was amplified by PCR from pBac{3×P3-DsRedaf} with the primer pair pDsRed-f (5′-ATGGTGCGCTCCTCCAAGAACGT-3′) and pDsRed-r (5′- CAGGAACAGGTGGTGGCG-3′).

**Table 2:** Identification of the genomic insertion sites of the pBac{3×P3-DsRed; FRT-3×P3-EGFP-SV40-FRT} vector by inverse PCR.

Strain	Scaffold	Chromosome	5′-Genomic sequence	3′-Genomic sequence
TTS-1	nscaf3026	23	CTTAAATAATTTAGTTTTCT**TTAA**	**TTAA** TAAGCTTGGGCATCTGTATA
TTS-2	nscaf2902	18	TGAATGTCAGAAAAACATGC**TTAA**	**TTAA** ATGCACAGATGGGTGCACAA

The flanking genomic sequences obtained with insertion site TTAA on the *piggyBac* left arm and *piggyBac* right arm are shown separately as 5′ and 3′ Genomic sequence. Localization of the silkworm genomic insertion sites of the pBac{FRT-3×P3-EGFP-SV40-FRT} vector was completed using the SilkMap application (www.silkdb.org/silksoft/silkmap.html).

These two PCR products were subjected to electrophoresis and recovered from the gel. Both fragments were labeled with DIG-High Prime reagent from the DIG High Prime DNA Labeling and Detection Starter Kit II (Roche, Mannheim, Germany). The DNA samples on the membrane were prehybridized for 1 h at 68°C, and hybridized overnight with the DIG-labeled EGFP probe and DsRed probe. The membrane was washed twice in 2×SSC containing 0.1% SDS for 15 min, and then washed twice at 65°C in 0.1×SSC containing 0.1% SDS for 15 min each time.

The detection of hybridized DNA was done using a chemiluminescent method with ready-to-use CSPD (Roche, Mannheim, Germany) according to the manufacturer’s instructions. The blotting results were observed using a chemiluminescence imaging system (Clinx ChemiScope3400 Mini, Shanghai, China).

**Table 3:** Injection of FLP recombinase expression vector into silkworm embryos obtained by crossing heterozygous G1 TTSs males with wild-type females.

Crossing(♂×♀)	Injectedvector(ng/µL)	NumberofInjectedeggs	Number ofhatchedeggs (%)	Numberof totalG2 fertilemoths	Numberof DsRedand GFP-positiveG2 fertilemoths	Number oftotal G3broods withDsRed andGFP-positivelarvae	Number ofG3 broodswithcontainsonly DsRed-positivelarvae	Number ofonly DsRed-positive G3larvae inthe broods	Recombinationfrequency %*
TTS-1 ♂×wild-type ♀	pSLA3-FLP(325 ng/µL)	524	246 (46.95%)	186	98	85	3	18	3.53
TTS-2 ♂×wild-type ♀	pSLA3-FLP(325 ng/µL)	463	187 (40.39%)	147	76	58	2	14	3.45
Total		987	433 (43.87%)	333	174	143	5	32	3.5

* Percentage of (Number of G3 broods with contains only DsRed-positive larvae)/(Number of total G3 broods with DsRed- and GFP-positive larvae).

## Results

### Experimental Design

The method of deleting the target gene using the FLP/*FRT* site-specific recombination system in silkworm involves the following steps, as illustrated in Figure 1: (1) Genomic loci were tagged by pBac{FRT-3×P3-EGFP-SV40-FRT} vector (Figure 1A) insertions through the *piggyBac*-mediated germline transformation of diapause silkworm strains [38], resulting in the production of stable G1 TTSs (Figure 1C); (2) The FLP recombinase-expressing helper vector pSLA3-FLP (Figure 1D) was microinjected into heterozygous G2 TTS embryos (Figure 1E). Site-specific recombination between two *FRT* sites of the TTS genome was mediated by the FLP recombinase expressed by the helper vector pSLA3-FLP, resulting in the deletion of the 3×P3-EGFP expression cassette in G3 SSRSs (Figure 1F). (3) On the basis of the different fluorescence phenotypes for either site-specific recombinant or non-site-specific recombinant silkworms, site-specific recombinant-positive individuals were screened using fluorescence microscopy. The TTSs contained a 3×P3-DsRed expression cassette and a *FRT*-flanking 3×P3-EGFP expression cassette, whereas the SSRSs should have only the 3×P3-DsRed expression cassette after the site-specific deletion of 3×P3-EGFP between the two *FRT* sites in the TTSs genome. Therefore, recombination of the two *FRT* sites would result in loss of green fluorescence but retention of red fluorescence within the eyes and nervous system of the silkworms.

**Figure 3:**
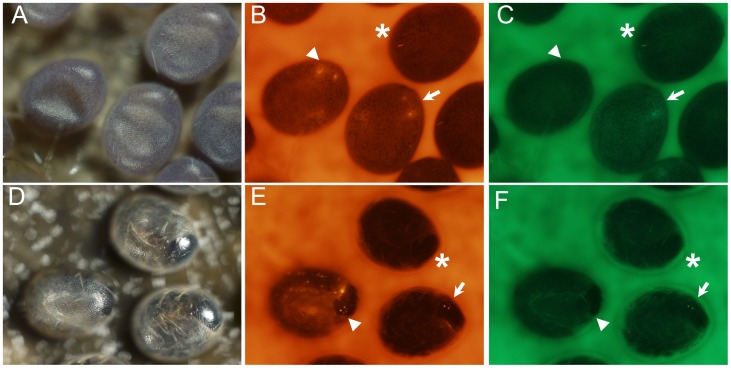
Expression of the DsRed and EGFP genes detected at different developmental stages of G3 silkworm individuals. (A–C) show white light (A), RFP-fluorescent (B) and GFP-fluorescent (C) images of 6-day-old G3 silkworm embryos. (D–F) show white light (D), RFP-fluorescent (E) and GFP-fluorescent (F) images of the 7-day-old G3 silkworm embryos. The DsRed- and GFP-positive non-site-specific recombinant transgenic embryos are highlighted with an arrowhead; DsRed-positive site-specific recombinant transgenic embryos are highlighted with a triangle, and wild-type embryos are indicated with an asterisk.

**Figure 4:**
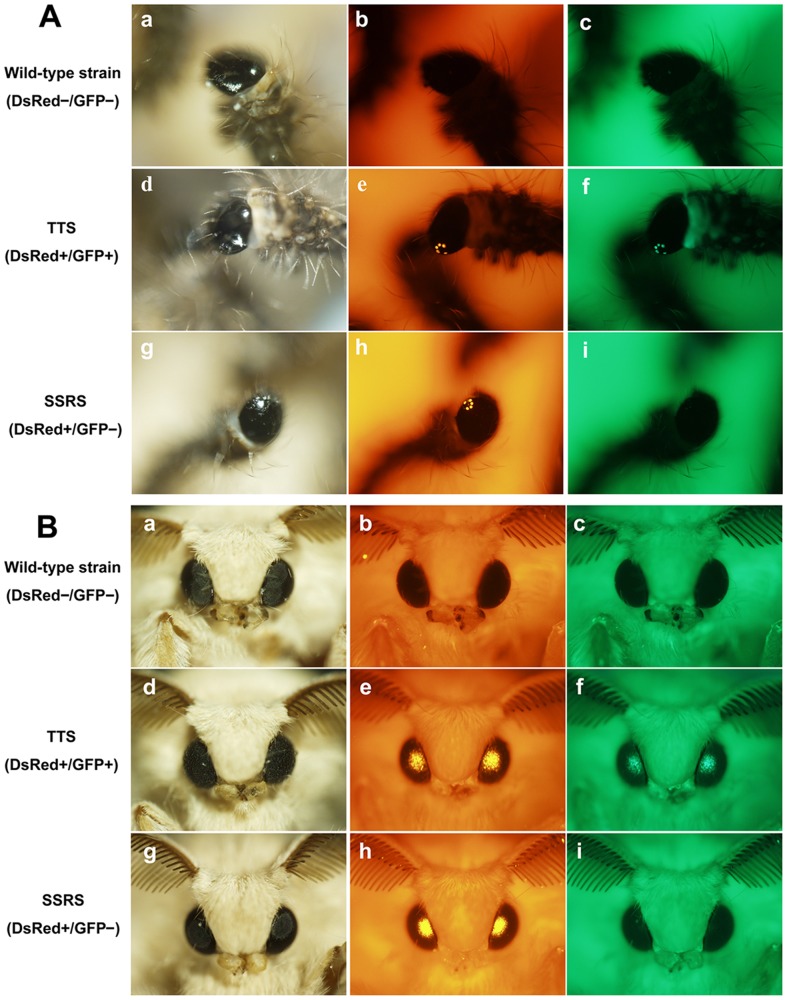
Expression of the DsRed and EGFP genes in larvae and adults from TTS and SSRS silkworms. (A) The newly hatched larvae of wild-type strain (a–c), TTS-1 (d–f) and SSRS-1 (g–i) showing white light (a,d,g), RFP fluorescence (b,e,h) and GFP fluorescence (c,f,i) in the developing larval ocelli. (B) The adults of the wild-type strain (a–c), TTS-1 (d–f) and SSRS-1 (g–i) showing white light (a,d,g), RFP fluorescence (b,e,h) and GFP fluorescence (c,f,i) in the compound eye.

### Production of TTSs for Silkworm FLP Recombinase-mediated Site-specific Recombination

To create stable silkworm TTSs containing *FRT*-flanked 3×P3-EGFP expression cassettes, 330 G0 non-diapause eggs from the wild-type *B. mori* strain Dazao were microinjected with the pBac{3×P3-DsRed; FRT-3×P3-EGFP-SV40-FRT} vector and helper plasmid pHA3PIG mixture. G0 adults were mated with each other or backcrossed with the wild-type Dazao strain. In total, we obtained 28 G1 broods, including two broods that had at least one DsRed- and one GFP-positive larva (Table 1). The percentage of G1 broods with DsRed- and GFP-positive larvae was 7.14%. G1-positive individuals from the two broods were reared (each brood was a unit), and 28 G1 DsRed- and GFP-positive individuals were obtained. The fluorescence images of a positive individual are shown in Figure 2. Finally, we established two stable G1 FLP/*FRT* system TTSs, which were named TTS-1 and TTS-2.

**Figure 5:**
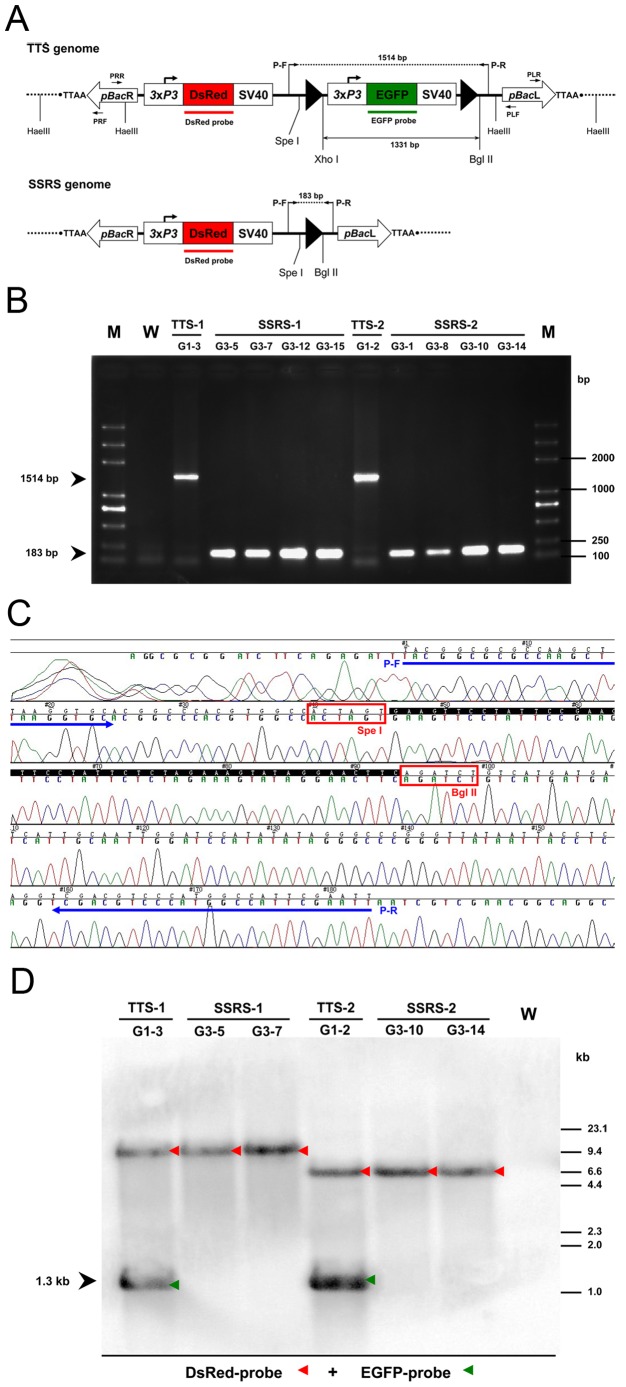
Molecular confirmation of FLP-mediated site-specific recombination in silkworm. (A) Schematic map of the FRT-3×P3-EGFP-SV40-FRT cassette before (*top*) and after (*bottom*) site-specific excision in transgenic silkworms. Two *FRT* sites (*black triangles*) before (*top*) and after (*bottom*) recombination are flanked by different restriction sites. The *Spe*I, *Xho*I and *Bgl*II are shown, as are the recognition sites of P–F/P–R primer pair used for PCR analysis. A 1514-bp amplicon was detected from the G1 TTS genome (*top*) and a 183-bp amplicon was detected from the G3 SSRS genome (*bottom*). *Xho*I- and *Bgl*II-digested genomic DNAs were hybridized to DsRed and EGFP probes. The EGFP probe hybridized fragment size calculated before recombination (*top*) was 1331 bp. *Hae*III-digested genomic DNAs were used to make templates for inverse PCR. PLF/PLR and PRF/PRR are the *piggyBac* left and right arm (*pBac*L and *pBac*R) primer pairs, respectively. (B) PCR confirmation of FLP recombinase-mediated site-specific excision in G3 transgenic silkworms. Genomic DNAs from two adults of G1 TTSs and eight adults of G3 SSRSs were used as DNA templates for PCR to confirm excision of the *FRT*-flanked 3×P3-EGFP expression cassette with primers P–F/P–R. Wild-type silkworm (W) were used as a control. Lane M, the Trans2K Plus DNA Marker. (C) Sequencing result of the 183-bp PCR products from all SSRS-positive individuals. Horizontal arrows show the primer pair P–F/P–R. Black background sequence shows a 48-bp recombinant *FRT* site in the genomic DNA. (D) Southern blotting analysis of FLP recombinase-mediated site-specific recombination. 25 ug genomic DNA samples were digested with *Xho*I and *Bgl*II, separated by agarose gel electrophoresis, and hybridized with *DsRed* and *EGFP*-specific probes. The individual DNA hybridization patterns of the wild-type (W), TTS-1 (G1–3), SSRS-1 (G3–5, G3–7), TTS-2 (G1–2) and SSRS-2 (G3–10, G3–14) lanes are shown. Red triangles and green triangles denote the signals for the DsRed and EGFP probes, respectively.

Genomic DNA was extracted from TTS adults, and inverse PCR analyses were performed to determine the insertion position and copy number of the transgene construct in individuals from TTS-1 and TTS-2. The inverse PCR results showed that each TTS adult contained only one copy of the transgene construct (data not shown). The silkworm genomic sequences flanking the *piggyBac* arms are shown in Table 2. The comparison of these sequences in the SilkDB showed that all of them fullly matched contig sequences in the database. Two TTSs carried the transgene in a heterozygous state. The inserts of *piggyBac* in the genome of TTS-1 and TTS-2 were located on chromosome 23 and 18, respectively.

**Table 4:** Comparison of the recombination efficiency mediated by the FLP/*FRT* system in other higher eukaryotes.

Species	Recombinasetype	Method for introducing FLP-expression	Target gene/Targetsequence	Use of FRTsite	Recombinationefficiency^4^	Reference
*Arabidopsis thaliana*	FLP	Cross	*β-glucuronidase* (GUS)	Gene inversion	20%	Sonti et al. [17]
*Oryza sativa*	FLP	Cross	*Neomycin phosphotransferase II*(NPTII)	Gene excision	∼25.6%	Hu et al. [4]
*Zea mays*	FLP	Cross	*Acetolactate synthase* (ALS)	Gene excision	40.7%	Li et al. [23]
*Nicotiana tabacum*	FLP	Chemically-induced	*Hygromycin phosphotransferase*(HPT); FLP	Gene excision	13–41%	Woo et al. [22]
*Drosophila* *mlanoguster*	FLP	FLP expression plasmidinjection	EYFP; ECFP	FLP-RMCE^3^	22–31%^5^	Horn et al. [28]
*Danio rerio*	FLPe^1^	FLPe mRNA injection	*mylz2*-EGFP	Gene excision	∼84%	Wong et al. [46]
*Caenorhabditis* *elegans*	FLP	FLP expression plasmidinjection; Heat shock-induced; Tissue-specificexpression	*unc-119p::unc-119*	Gene excision	Not given	Vázquez-Manriqueet al. [6]
*Xenopus laevis*	FLPe	FLPe cRNA^2^ injection	CarAct-eGFP; Rhodopsin-mCherry; Six6-mCherry	FLP-RMCE	∼25%	Zuber et al. [47]

1 FLPe, a thermostable FLP mutant.

2 cRNA, complementary RNA.

3 FLP-RMCE, FLP recombinase-mediated cassette exchange.

4 Efficiency of recombinant individuals in F1 transgenic plants or G0 injected transgenic animals somatic cells except *Drosophila mlanoguster*.

5 Percentage of (F1 crosses with at least one recombinant offspring)/(fertile F1 crosses).

### Production and Analysis of Site-specific Recombinants

To explore the feasibility and efficiency of FLP recombinase-mediated site-specific recombination in silkworm, heterozygous G1 male adults from each of the two TTSs were backcrossed to wild-type female adults to produce G2 non-diapause embryos for helper plasmid pSLA3-FLP microinjection. In total, 987 G2 embryos were injected, and 174 G2 DsRed- and GFP-positive adults were obtained. To screen for the individuals with germline site-specific recombination, 174 G2 DsRed- and GFP-positive adults were backcrossed with those from the wild-type Dazao strain and the 143 G3 broods obtained were analyzed for fluorescence phenotypes (Table 3). Finally, 32 G3-positive recombinant embryos with only RFP fluorescence were obtained from five broods among the 143 G3 DsRed- and GFP-positive broods (Figure 3). The *piggyBac* transposon-derived construct in the genome of two TTSs were both single copies; the average frequency of FLP recombinase-mediated site-specific excision in two TTSs was 3.49%. Figure 4 shows the expression of the DsRed and EGFP genes in larvae and adults of TTS-1 and SSRS-1 silkworms.

To confirm FLP recombinase-mediated site-specific excision of positive recombinant individuals, PCR was performed on genomic DNAs from G1 TTS adults, G3 SSRS adults and wild-type adults with the primers P–F and P–R (Figure 5A). As shown in Figure 5B, the PCR products were a 1514-bp DNA fragment for two individuals of G1 TTSs, a 183-bp DNA fragment for eight individuals of G3 SSRSs and no amplified fragment for wild-type silkworm control, which is consist with the putative pattern. The 183-bp PCR products from all G3 SSRSs positive individuals were sequenced (Figure 5C). These results also verified that the precise site-specific recombination between two *FRT* sites in the genome of these TTSs individuals was mediated by FLP recombinase.

The results of site-specific recombination were also confirmed by Southern blotting analysis with DsRed and EGFP probes (Figure 5A). Genomic DNAs were obtained from G1 TTS adults, G3 SSRS adults and wild-type adults and fully digested with *Xho*I and *Bgl*II (Figure 5A). The blotting results presented two bands in the samples from G1 TTS individuals, which is consistent with the expected band pattern of a 1.3-kb band blotted by the EGFP probe and another band blotted by the DsRed probe. The samples from G3 SSRS individuals all showed only one band blotted by the DsRed probe, and those derived from the same TTS had the same size of band blotted by the DsRed probe. Thus, the Southern blotting results not only showed that the blotting band pattern is identical to the expected band pattern, but also confirmed that individuals from each TTS contained a single copy of the transgene construct as described above.

## Discussion

The results reported herein provide the first demonstration of the use of the FLP/*FRT* system in the genetic manipulation of the silkworm genome. Pre-blastoderm microinjection of an FLP helper vector resulted in the deletion of the *FRT*-flanked target gene in genome of TTS offspring. The average frequency of FLP recombinase-mediated site-specific excision in two TTSs was approximately 3.5%. The recombination efficiency was lower compared with reported numbers from other higher eukaryotes (Table 4). One possible reason is the high efficiency of the transient expression of the FLP gene in silkworm eggs at the pre-blastoderm stage, which can increase the recombination efficiency by FLP recombinase-mediated expression in the silkworm. In our experiment, a 0.65-kb truncated silkworm A3 promoter [41] was used to regulate the transient expression of the FLP gene in the pre-blastoderm of eggs from TTSs. It has been reported that the truncated silkworm A3 promoter had an approximately 20-fold increase in promoter activity in transient expression assays compared with the wild-type silkworm A3 promoter [44]. Another possible reason for the low recombination efficiency is that only a small amount of mature and activated FLP proteins derived from helper vector pSLA3-FLP is produced in silkworm eggs at the pre-blastoderm stage. The union of male and female silkworm gametes forms a zygote approximately 2 h after oviposition, and the zygote splits to form the blastoderm 13 h after fertilization [45]. During the embryonic development of silkworm, karyokinesis occurs first, then followed by cytokinesis [45]. The germ cells of the adult silkworm are derived from primordial germ cells during early embryo development stages [45]. Thus, the stable SSRS individuals might have been produced only when these site-specific recombination events had occurred in the primordial germ cells. In this experiment, a large amount of mature and activated FLP proteins derived from pSLA3-FLP might have been produced after blastoderm formation, resulting in a low frequency of positive site-specific recombinant offspring. To accelerate FLP protein aggregation in the pre-blastoderm of silkworm eggs, FLP mRNA can be injected into the embryos of TTSs to direct FLP recombinase synthesis. At present, this method has only been reported in some higher model organisms such as zebrafish (*Danio rerio*) [46] and *Xenopus laevis* [47], showning a high recombination efficiency in somatic cells of transgenic zebrafish (Table 4). Although a low efficiency of FLP/*FRT* system-mediated site-specific gene excision was obtained in the current study, the recombination efficiency was similar to the *piggyBac*-mediated transgenic germline transformation of the silkworm [38], [48], which is the most conventional transgenic methodology for this species. These data also suggest that the FLP/*FRT* system is a potentially useful tool for the site-specific integration or knockout of transgenes in the silkworm.

In our study, a direct injection method rather than sexual hybridization method was used to introduce and express the FLP gene in the pre-blastoderm of eggs from TTSs. The main disadvantage of the sexual hybridization method is that the FLP gene sequence would be introduced to the genome of the hybrid offspring, and unless it is crossed out, persistent FLP expression could negatively affect the presence or function of the target or donor genes. Although there have been no reports of FLP toxicity *in vitro* or *in vivo*, the risk of FLP toxicity still cannot be completely ruled out from the persistent expression of the FLP gene in hybrid offspring. Moreover, the FLP recombinase-mediated site-specific excision reaction between two *FRT* sites is reversible [21]; thus, the persistent expression of the FLP gene might affect the recombination efficiency and the stability of the target site. The injected FLP helper vector is gradually degraded during embryonic development, thereby effectively avoiding occurrence of the above problems.

The FLP/*FRT* system-based site-specific recombination technology has been widely used in *Drosophila*. Similar to *Drosophila*, *B. mori* is also a model organism for studies of the genetics of higher eukaryotes. The recombination activity of FLP recombinase in cultured cells and embryos of *B. mori* was confirmed as early as 1999, and the extrachromosomal recombination efficiency was approximately 20% in *Bm*N4 cells and embryos [31]. In addition, FLP is a temperature-sensitive recombinase [49]. Compared with other site-specific recombination systems, the main advantage of using the FLP/*FRT* system in silkworms is that the optimum temperature of the FLP recombinase (30°C [50]) is closer to the embryonic development temperature of *B. mori* (approximately 25°C). The sophisticated Cre/*loxP* system is the most widely applied tool for genome manipulation at present, but the optimum temperature of Cre recombinase (37°C [50]) is higher than the embryonic development temperature of *B. mori*. Thus, the Cre/*loxP* system is probably more suitable for genetic manipulation in mammals [50]. Therefore, the FLP/*FRT* system was selected for *B. mori* site-specific recombination in this study.

Strategies for genetic engineering generally require stable and inheritable modification of the target genome. Most methods for the introduction of exogenous DNA into the target genome are characterized by random integration. Random insertion mediated by transposons is particularly efficient in many different organisms, but the lack of control at the introduced DNA position leads to unpredictable variations in gene expression and undesirable mutagenesis of important genes. A germline transformation method using the *piggyBac* transposon as a vector has been developed to create transgenic silkworms [41], [48], [51], [52]. Although this method is reproducible and reliable, the *piggyBac*-mediated random insertion of exogenous genes into the host animal genome still cannot be overcome [41], [51]. In addition, it has been reported that integrated *piggyBac* elements could be remobilized in the genomes of *D. melanogaster*, the beetle *Tribolium castaneum*, the mosquito *Anopheles stephensi* and *B. mori* [53]–[56]. During the large-scale rearing of the commercial transgenic silkworm strains, we also observed the phenomenon of *piggyBac* transposon remobilization (data not shown). Based on the results of our study, after the specific acceptor sites (*FRT* sites) have been generated by *piggyBac*-mediated transgenesis and suitable loci have been identified in silkworm, the introduction of transgenes by FLP recombinase-mediated site-specific recombination will be easy to generate and examine. By removing one or both of the two terminal sequences of the *piggyBac* transposon after integration, the insertion can be stabilized [57], [58]. This strategy can not only eliminate undesirable transgene expression that results from *piggyBac*-mediated random insertions into the silkworm genome, but also minimize the position effect in silkworm functional genomics research by means of creating and selecting appropriate TTS for subsequent germline transformation. Thus, once an appropriate and stable TTS with no or one of two terminal sequences of the *piggyBac* transposon after integration is established, this system would be a good candidate for site-specific transformation of *B*. *mori*. Furthermore, controlling for position effects by FLP/*FRT* system-based genomic targeting will also enable optimization of heterologous protein expression in *B. mori* for use as a protein bioreactor [40], [59], [60]. In the future work, FLP-RMCE will be introduced as a powerful tool for site-specific gene targeting in silkworm.

In conclusion, this study is the first to demonstrate the feasibility of FLP recombinase-mediated site-specific recombination for *B*. *mori* genome manipulation. Our experiments have a huge improvement for *B*. *mori* genome manipulation using the FLP/*FRT* system since our experiments first obtained stable germline transformation in the individual level rather than the cell and tissue levels reported by Tomita et al, which only obtained the extrachromosomal (plasmid-based) site-specific excision [31]. Our results are likely to accelerate the practical application of the FLP/*FRT* system in the genomic manipulation of silkworm and promote the establishment of a FLP/*FRT* system-based research platform for the functional analysis of unknown genes in silkworm. Moreover, the significance of this work is not confined to studies of silkworm functional genomics, but would also be relevant for the practical utilization of silkworm transgenic lines in sericulture, silkworm bioreactors and silkworm modeling.
